# Edge Computing of Online Bounded-Error Query for Energy-Efficient IoT Sensors

**DOI:** 10.3390/s22134799

**Published:** 2022-06-24

**Authors:** Ray-I Chang, Jui-Hua Tsai, Chia-Hui Wang

**Affiliations:** 1 Department of Engineering Science and Ocean Engineering, National Taiwan University, No. 1, Sec. 4, Roosevelt Road, Taipei 10617, Taiwan; rayichang@ntu.edu.tw (R.-I.C.); r02525059@ntu.edu.tw (J.-H.T.); 2 Department of Computer Science and Information Engineering, Ming Chuan University, No. 5 Der-Ming Rd., Gwei Shan District, Taoyuan City 333, Taiwan

**Keywords:** internet of things, wireless sensor networks, bounded-error, query processing, online query, energy efficient, edge computing

## Abstract

Since the power of transmitting one-bit data is higher than that of computing one thousand lines of code in IoT (Internet of Things) applications, it is very important to reduce communication costs to save battery power and prolong system lifetime. In IoT sensors, the transformation of physical phenomena to data is usually with distortion (bounded-error tolerance). It introduces bounded-error data in IoT applications according to their required QoS^2^ (quality-of-sensor service) or QoD (quality-of-decision making). In our previous work, we proposed a bounded-error data compression scheme called BESDC (Bounded-Error-pruned Sensor Data Compression) to reduce the point-to-point communication cost of WSNs (wireless sensor networks). Based on BESDC, this paper proposes an online bounded-error query (OBEQ) scheme with edge computing to handle the entire online query process. We propose a query filter scheme to reduce the query commands, which will inform WSN to return unnecessary queried data. It not only satisfies the QoS^2^/QoD requirements, but also reduces the communication cost to request sensing data. Our experiments use real data of WSN to demonstrate the query performance. Results show that an OBEQ with a query filter can reduce up to 88% of the communication cost when compared with the traditional online query process.

## 1. Introduction

The sensors of IoT (Internet of Things) applications sense, collect, and transmit important data from their surroundings. The large amount of information among billions of sensors creates massive energy consumption. Visionary green IoT [1] reduces the energy consumption of sensors and makes the environment safer. The latest hardware advances such as [2,3,4,5] in the unprecedented development for IoT and AI (artificial intelligence) have led to smart edge devices, such as smartphones, smartwatches, and smart glasses, which can sense and think. Due to the hardware breakthroughs in new materials and nano-devices, in-memory computing and sensing clearly resolve the energy and time bottlenecks incurred from the sequential digitization of analog sensory signals.

However, IoT wireless sensors might spend up to 90% of their power on communication [6]. Hence, reducing communication costs is crucial for saving battery power and prolonging the system lifetime. In IoT sensors, the transformation of physical phenomena to data is usually with distortion (bounded-error tolerance). For instance, data distortion is caused by hardware [7] and ADC (Analog-to-Digital converters) transformation [8]. Hence, data distortion is usually set within an acceptable range in a system design. For example, the National Taiwan Central Weather Bureau accepts a 0.1 °C bounded-error in temperature [9,10]. It introduces bounded-error data in IoT applications according to their required QoS^2^ (quality-of-sensor service) or QoD (quality-of-decision making).

In bottom-up WSN (wireless sensor network) architecture, our previous work [11] proposed a bounded-error data compression scheme called BESDC (Bounded-Error-pruned Sensor Data Compression) to reduce the communication cost dramatically. In this paper, we couple edge computing and bounded-error sensor data compression to propose an online bounded-error query (OBEQ), which can further reduce communication costs. We use the bounded-error as an artifice for reducing the communication cost of top-down query processes in WSN, without affecting the demanding qualities of sensor service and decision making (respectively known as QoS^2^ and QoD) in IoT applications.

In Section 2, recent bounded-error-related methods and our unique bounded-error definition are introduced. In addition, the online query mechanism in wireless network techniques is discussed. In Section 3, an OBEQ is proposed and depicted in detail. In Section 4, OBEQ evaluations are presented. The last section summarizes the proposed OBEQ solution and future work.

## 2. Related Works

In WSNs, a query process for sensor data can be an offline query or an online query [12]. An offline query is a gather-then-query method and an online query is a query-then-gather method for the user. In online query processes, a user can query current sensor data by sending a query to a local database on the network edge of a WSN, and then the edge-computing database will inform the WSN to return queried data. In a WSN, each wireless sensor executes a query for raw sensor data and returns the query results. In most cases for IoT applications, sensor errors are considered as noise. However, allowing error bounds in WSN sensor data can be a valuable artifice to solve certain problems. For example, in 2003, a TiNa scheme [13] used the error bound between former sensed data and current sensed data to decide the transmission of current data, and thus reduce the communication cost of wireless nodes.

In 2012 and 2020, refs. [11,14] applied bounded error techniques, respectively, to improve data compression ratios by applying error bound on raw sensor data during different compressions. The sensors’ power consumption in data communication could be reduced, thus prolonging the IoT’s system lifetime. In 1997 and 2013, both [15,16] also used bounded error to reduce the response time of aggregation functions in query language for a large database. These bounded-error compression techniques can be cost-effectively applied in offline queries to reduce the data communication cost to the local database for gather-then-query. However, since IoT sensor networks have grown much bigger than ever, offline queries are not suitable for large-scale networks without sufficient storage in local databases. Previous online query methods usually ignore the error of data and cannot manage bounded-error data accurately. Currently, there is no query engine available for bounded-error requirements of data engineering.

The most important mechanism in online query is the query processor in wireless sensors. Query processors need to translate queries into execution plans and eventually carry out the execution plans and generate query results [17,18,19]. In 2002, COUGAR was one of the first systems that supported the WSN online query [20]. A query layer was added to wireless sensors to answer simple SQL queries. The prototype of the online query in COUGAR can be traced back to the architecture in their previous work [21]. At almost the same time, another WSN DB called TinyDB [22] was proposed. TinyDB provides a simple SQL query interface and has a distributed query processor running at each node in the WSN. Users can submit queries in a base station and then the query will be optimized and sent to the WSN [23]. However, none of the above WSN query techniques consider the bounded-error feature of raw sensor data.

**Figure 1:**
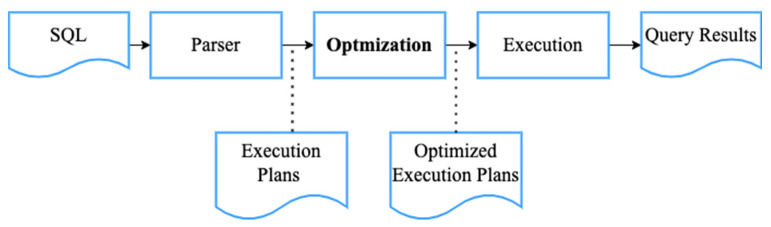
The flowchart of an SQL query process.

As shown in Figure 1, SQL is parsed into a syntactic tree at the beginning of the database query. Then, the syntactic tree is compiled into execution plans, which contain deeper information on databases, tables, and other metadata. After execution plans are generated, the optimizer arranges the tree to optimize the performance. In the final step, execution plans are carried out and generate a query result. SQL includes data definition language (DDL) and data manipulation language (DML) [19]. DDL describes the record, fields, and sets of the user data model. It connects the conceptual level and internal level. CREATE, ALTER, and DROP are the three main syntaxes of DDL. In IoT, each wireless sensor can function as a distributed micro database [24]. Micro databases not only store data at the internal level, but they are also strictly connected to a sensor module. DDL is extended to influence the sensor module. This enables users to control sensor module flexibly and manage the hardware resources. The DML allows users to query the database and SELECT-FROM-WHERE-GROUPBY is the main structure of DML [19].

## 3. Online Bounded-Error Query (OBEQ)

In this paper, an SQL-like language [25,26] is used to depict the required data for the bounded-error query and our BESDC [11] is applied for returning the query results from each sensor to reduce the communication cost to prolong the WSN’s system lifetime. An OBEQ is designed to prune the sensor data with bounded-error to further maximize the compression ratio for the system lifetime extension. In addition, an OBEQ applies BESDC to further reduce the bottom-up communication cost from sensors to the local edge-computing database, after the top-down query was issued from the user to the sensors. Since users can specify a bounded-error range on each query, the worst case of data distortion is evaluated to be controlled. We can guarantee that the error would not exceed the user-specified bounded-error. Assume that the user-specified bounded-error is larger than the WSN’s system error; if this system error is bigger than the user’s requirement of QoS^2^ and QoD, then the deployed WSN system is obviously not qualified for the user’s task.

**Figure 2:**
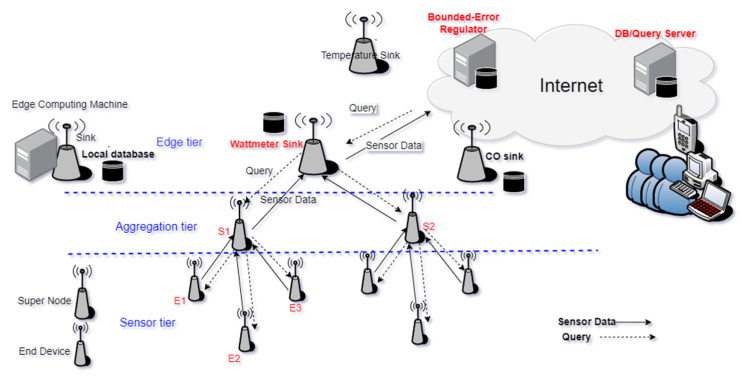
System architecture of an edge-computing OBEQ for IoT (S1, S2 are SNs, E1–E3 are EDs).

Our OBEQ system consists of a local database and a WSN. This WSN is composed of numerous end devices (ED)s deployed in surroundings to gather data periodically. The system architecture of an edge-computing OBEQ is illustrated as shown in Figure 2. It is deployed in a three-layer architecture. The first layer of the edge-tier consists of sink nodes, which have stable power and communication resources for Internet connections. Sinks are responsible for communicating between other layers of WSN and local databases. The second layer of the aggregation-tier consists of super nodes (SN). SNs are responsible for transmitting information between the sink and their dominated EDs. SNs are also capable of aggregating, merging, and compressing query results. The third layer of the sensor layer consists of EDs, which are responsible for converting the status of the physical world to digital data periodically. EDs and SNs are also capable of executing bounded-error query processes and compression.

**Figure 3:**
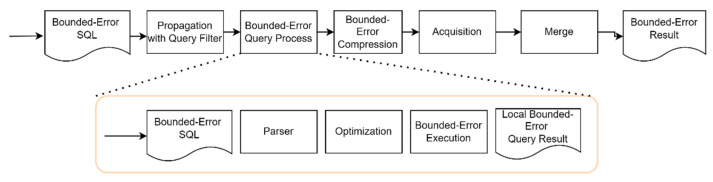
The flowchart of the OBEQ system.

In an OBEQ, bounded-error parameters are added to SQL, which allows users to set up a bounded-error range for a query result. For each query instruction, the change in bounded-error in each step in an OBEQ is examined. Figure 3 shows the flowchart of OBEQ’s bounded-error query process. The query result shown in the lower flowchart is the result generated by a single wireless sensor. It is not the final query result since the “Local Bounded-Error Query Result” is different to the final result of “Bounded-Error Result” in the upper flowchart. The complete query result is not finalized until all the EDs send their query results to the local database of the SNs to merge all sensor data. In our system, users query information through the local database. The local database analyzes the query instruction and propagates a query into the WSN. The instruction is sent through the sink and SNs before reaching the EDs. The EDs process the query and generate local query results. During the bounded-error query process, our system maximizes the compression error tolerated to achieve a better compression ratio. In the end, the compressed local query results are sent back through SNs, sinks, and the local database.

### 3.1. OBEQ Query Filter

The transmitting of data is extremely power consuming for wireless sensors. At the beginning of the bounded-error query process, the local database uses sinks to propagate query instruction to its WSN. However, for some queries, it is not necessary to access all the SNs and EDs. A location-based query is one example. When we query only some data from sensors in the “Taipei city” sink, the query should not be sent to sensors in the “New Taipei city” sink. The query filter is proposed to avoid redundant data transmission by analyzing the WHERE clause of the SQL query.

Since SN is responsible for transmitting query instructions to particular sets of EDs, the EDs under one SN contain three situations to be judged by the relation between the WHERE clause in the SQL query and the metadata of EDs.

1. All EDs under the SN violate the WHERE clause in a query instruction.
2. Part of the EDs under the SN violate the WHERE clause in a query instruction.
3. All EDs under the SN are qualified by the WHERE clause in a query instruction.

Therefore, when an SN detects the first situation, the query instruction is discarded. When an SN detects the second situation, the query instruction should be sent to confirm that the target sensors, which are specified in the WHERE clause, will receive the query. For example, a query asks for water-meter data because the EDs under this SN are composed of both watt-meters and water meters. When an SN detects the third situation, the WHERE clause that all EDs are qualified to be acknowledged will be truncated because all EDs should execute the query. For example, a worker of the Taiwan Power Company wants to query all the power data of Taipei city. If all EDs under the SN are in Taipei city, then the “WHERE city = Taipei” clause in the SQL instruction is removed by the SN, and the rest of the query instruction is transmitted.

In the following equations, $ED_{WHERE}$ represents all the EDs that are qualified in the constraint of the SQL WHERE clause, $SN_{i}$ represents *SN_i_*, $ED_{SNi}$ represents all the EDs under $SN_{i}$, $QL_{rd}$ is the bit length of the query instruction $SN_{i}$ received, and $QL_{st}$ represents the bit length of the query instruction after $SN_{i}$ truncates the original query with the query filter. When Equation (1) is true, $SN_{i}$ abandons the query instruction without forwarding to its EDs. When Equation (2) is true, $SN_{i}$ truncates the constraints specified in the WHERE clause to reduce the length of the query instruction forwarded to its EDs. Therefore, $QL_{rd}$ is not smaller than $QL_{st}$ as shown in Equation (3).

(1) $$ED_{SNi}\cap ED_{WHERE}=\emptyset$$

(2) $$ED_{SNi}\subseteq ED_{WHERE}$$

In addition, $Cost_{no\text{-}filter}$ is used to represent the energy cost of a cluster of EDs under an SN without using a query filter, pw represents the power usage for sending a bit, and $Cost_{filter}$ is the energy cost of using a query filter. The cost of sending a filter-processed query packet and no-filter one can be deduced as follows.

(3) $$QL_{rd}\geq QL_{st}$$

(4) $$Cost_{no\text{-}filter}=QL_{rd}\times pw$$

(5) $$Cost_{filter}=QL_{st}\times pw$$

(6) $$∴Cost_{no\text{-}filer}\geq Cost_{filter}$$

Thus, certain metadata [27] should be collected to judge whether a node violates a WHERE constraint or not. The metadata applied in our query filter with the ED_CLUSTER_METADATA clause is shown in Table 1. To judge if EDs (usually, a cluster of EDs under an SN) are in certain region, our query filter extracts the location from E the D_CLUSTER_METADATA clause and then compares the location within the constrained area specified in the query instruction. Note that each SN must record the locations of its EDs. In addition, an SN has to record the deployed time of EDs. When a query filter judges that EDs are qualified for a specific time interval, the deployed time is extracted and compared with the time interval in the query instruction. The same idea can be applied to sensor types and attribute columns.

### 3.2. Bounded-Error Query Processing

The bounded-error query process designs an SQL-like language, which is constructed by the SELECT-FROM-WHERE-GROUPBY structure and bounded-error parameter. The bounded-error of each sensor’s data is guaranteed to be limited in the user-specified range of his/her query. DDL depicts the record, fields, and sets of the user data model and connects the conceptual level and the internal level of the database. CREATE, ALTER, and DROP are the three main syntaxes of DDLs. In an OBEQ, DDL is extended to sensor modules and enables users to control sensor modules’ flexibly to manage hardware resources in EDs.

**Figure 4:**
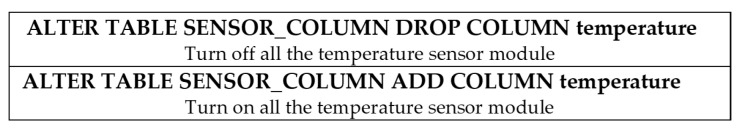
Syntactic examples of DDL.

In real applications, the user will need to modify the database constantly due to newly sensed data. However, it is resource-consuming for sensors to keep collecting suspended data while the newly sensed data are redundant. For example, some electric services provided by Taiwan Power Company [28] are suspended due to a user’s request. Hence, it is necessary to turn on/off the running sensor modules according to the database columns. To determine the relationship between the table columns and sensor modules, the edge-computing local database stores a SENSOR_COLUMN table. Table 2 shows an example of a SENSOR_COLUMN table and depicts the status of modules on wireless sensors. The corresponding syntactic examples of DDL are shown in Figure 4.

While the user sends a DDL instruction to the local database, it is separated into two instructions, one for offline databases and the other for online wireless sensors in the WSN. For example, “ALTER TABLE SENSOR_COLUMN DROP COLUMN current” is sent to modify the offline database directly. For WSN, “ALTER TABLE SENSOR_COLUMN” is redundant for wireless sensors because every ED needs to respond to this instruction. To reduce the transmission energy, edge-computing local databases can truncate “ALTER TABLE SENSOR_COLUMN” for its SNs and EDs before sending it to WSN. When an online query instruction arrives at the EDs, it will check the MODULE_COLUMN table, as shown in Table 3, to turn on/off the sensor and then gather corresponding information so that this table can be initialized before the sensors are deployed in the WSN.

As shown in Figure 3, the flowchart indicates that users first issue a query with bounded-error parameters. Then, this query is analyzed before being transmitted to the WSN. When a wireless device of either an SN or ED receives a query, it starts to process the query and generates a local query result. Then, finally, the local query results are sent to the local database and merged at the sink. Each sensor data error in a query result is bounded in the range specified by the user from an OBEQ. Meanwhile, one important feature of an OBEQ is that the applied bounded-error compression becomes better as bigger bounded-errors are assigned. Hence, it is important to maximize the bounded-error without damaging the QoS^2^ and QoD required for IoT applications.

In our modified SQL instructions, “.be(*τ*)” is used to set up the bounded-error for a query result. For example, the query instruction “SELECT Data.be(*τ*) FROM SENSORS” allows the query result to have an error no larger than *τ*. The results of query example “SELECT voltage.be(2) FROM SENSORS” are shown in Table 4. It shows the relation of raw data, physical data, and output query results for voltage data in EDs. The query result is allowed to have data distortion no bigger than two. Our OBEQ confirms that the worst scenario of data distortion will not exceed the specified bounded-error range. We assume that a value of 116 is the value from an ED and the exact value of physical data is 116.5, any value between 116.5 ± 2 (e.g., 117) is an acceptable query result.

Again, we use “SELECT Data.be(*τ*) FROM SENSORS” as an example for an ED to sense raw data according to the query. The raw data sensed by nodes have already shown a system error $\tau_{s}$ (see Table 3) due to sensor accuracy. After a local query result is generated, it needs to be compressed before sending tit o the SN for reducing power consumption. The compression for sensor output can be completed in the specified bounded-error $\tau_{c}$ without violating the QoS^2^/QoD required in diversified IoT applications. So, eventually, when the local query results are all sent to the local database, the total error *τ* of the query result is equal to $\tau_{s}$ plus $\tau_{c}$, as shown in Equation (7). The value of system error $\tau_{s}$ is related to the hardware equipment the user deploys in the WSN. Since the hardware has been settled, the value of $\tau_{s}$ remains unchanged during the query process. The error value $\tau_{s}$ can be found in the datasheet of the sensor. Since the bounded-error *τ* is usually given by users for the required QoS^2^/QoD in their IoT applications, the compression error $\tau_{c}$ can be arranged to further reduce the communication cost for query results. Finally, the compression error $\tau_{c}$ can be deduced as follows.

(7) $$\tau \geq \tau_{s}+\tau_{c}$$

(8) $$∴\;\tau_{c}\leq \tau -\tau_{s}$$

**Figure 5:**
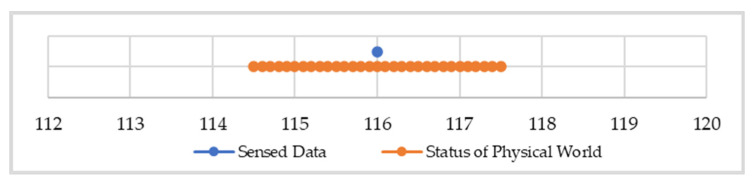
The bounded-error range of the raw data of voltage 116.

The SQL query of “SELECT voltage.be(2) FROM SENSORS” is used as a demonstration. In this query, voltage.be(2) indicates that all voltage data in the query result should have a data error lower than two. At the time that the data are sensed, a system error is added to a datum, as shown in Figure 5. The voltage in the physical world is a value between 116 ± 1.5 instead of 116 since the system error $\tau_{s}$ for voltage data is 1.5. The relationship between the raw data and the status of the physical world is shown in Figure 5. The upper bound and lower bound of the expected value of the status of the physical world are 114.5 and 117.5, respectively.

**Figure 6:**
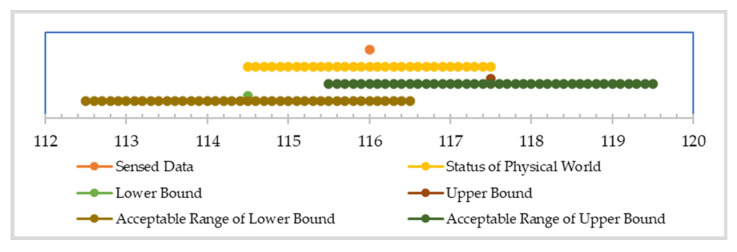
The range intersection of acceptable bounded-error data.

Because the worst scenario of data error is guaranteed to not exceed the user-specified bounded-error (i.e., *τ* = 2), the bounded-error data offered have to fully cover all the possible value that might happen in the physical world. In this example, if the upper bound is 117.5 for the raw data of voltage 116, according to the system error ($\tau_{s}$ = 1.5), then we can accept any sensor value between 115.5 and 119.5. Where the lower bound is 114.5, we accept any value between 112.5 and 116.5 because the user-specified bounded-error is 2. As shown in Figure 6, the intersection of the above two ranges is 115.5 to 116.5, which exactly equals 116 ± 0.5, where 0.5 equals the user-specified bounded-error value τ that subtracts the system error $\tau_{s}$. With the example described above, the error bound can be limited strictly as the user expects, and then the power consumption can be reduced further with our previous BESDC [11].

Our OBEQ method also supports addition, subtraction, multiplication, and division. It is a frequently used function in query processes. Parentheses and “.be()” are used to represent the error bound of a query result. For example, “SELECT (Data*m/*d* + *n*).be(*τ*)” indicates that the error of the calculated data should be limited in *τ*, where *m* stands for multiplicand and d stands for divider.

“SELECT (power/1000).be(1) FROM SENSORS” is an example to change the unit of the power column from W to kW. In this example, the bounded-error of the calculated result should not exceed 1 Wh. In WSNs, the calculations can be performed either in EDs or in the local database. How the calculations are performed by either an ED or a local database and how they influence the bounded-error are described below.

So, we use “SELECT (Data*m/*d* + *n*).be(*τ*)” as an example; if nodes are used to calculate the query result, following the principle of the four fundamental operations, the user-assigned error *τ* bounded for sensor data is related to (*m*/*d*)$\tau_{s}$ due to the multiplication and division with the system error $\tau_{s}$, then the error of the raw data stays (*m*/*d*)$\tau_{s}$ after addition and subtraction. After the query process and calculations are performed, the sensor has to compress the local query result before sending it to the SN. Using $\tau_{c}$ as a compression error, our sensor compresses the local query result and sends the compressed data that have bounded-error equal to (*m*/*d*)$\tau_{s}$ + $\tau_{c}$ to the local database. Hence, we can deduce that our $\tau_{c}$ can be calculated as follows.

(9) $$\tau \geq \tau_{c}+\frac{m}{d}\tau_{s}$$

(10) $$∴\;\tau_{c}\leq \tau -\frac{m}{d}\tau_{s}$$

If the local database is used to calculate the query result, the local query result that the sensors send to the local database would preserve the bounded-error ($\tau_{s}$ + $\tau_{c}$). In the local database, the local query result with error ($\tau_{s}$ + $\tau_{c}$) is calculated, and the error of the local query result becomes (*m*/*d*) ($\tau_{s}$ + $\tau_{c}$) after multiplication/division and stays (*m*/*d*) ($\tau_{s}$ + $\tau_{c}$) after addition/subtraction. The user expects (*m*/*d*) ($\tau_{s}$ + $\tau_{c}$) to be smaller than *τ*, so we can deduce the compression error as the following equations.

(11) $$\tau \geq \frac{m}{d}(\tau_{s}+\tau_{c})$$

(12) $$∴\;\tau_{c}\leq \frac{d}{m}\tau -\tau_{s}$$

Using “SELECT (power/1000).be(1) FROM SENSORS” as a demonstration, when EDs are assigned to do the calculation, each power datum has a system error equal to 0.5 (see Table 3). After the power is divided by a thousand, the system error becomes 1/2000. Then, we calculate the compression error and obtain 1999/2000. After compression, the error bound of the data becomes one to meet the user’s request and then the compressed data are sent to the local database. When the local database performs the calculation, sensors send the local query result with an error equal to (0.5 + $\tau_{c}$). The local database divides the local query results by one thousand, so now the error bound becomes (0.0005 + 0.001$\tau_{c}$), which should equal one. Hence, it can be calculated that the bounded-error for data compression to be used in this case is 999.5.

Considering the system error, the real status of the physical world is uncertain, yet can be deduced in a certain range. Using comparison operations, it is easy to judge that the status of the physical world is larger or less than a particular value if the possible range of the physical status is fully larger or less than that value. However, when the possible range covers the compared value, it is impossible to know whether the status of the physical world is larger or less than the value.

We describe three possible approaches to deal with it in data comparisons. Each proposed technique has a different syntax with unique advantages listed below.

**Syntax**: column_name<?value?
*The recall rate is maximized to 100% with a system error. If the range of the physical world covers the value, it is mapped into the query result.*
**Syntax**: column_name<“value”
*The precision rate is maximized to 100% with a system error. If the range of the physical world exceeds the value, it will not be mapped into the query result.*
**Syntax**: column_name<value
*The range of the physical world is treated as the value of raw data.*

The first syntax makes sure that all the data with system error $\tau_{s}$ and possibly smaller than the value are listed in the query result without false negatives in comparison. The second syntax makes sure that each datum in the query result is strictly smaller than the value, since the system error $\tau_{s}$ may cause false positives in comparison. The third syntax maps the query result by comparing the sensed data with the value without considering the system error.

For example, “SELECT node FROM SENSORS WHERE power < 100” queries nodes whose power usages are with a value smaller than 100. The system error of power according to Table 3 is 0.5. So, when raw data are 100, the physical status is the range between 99.5 and 100.5. Considering the system error, with this kind of query, it is difficult to determine whether the power usage of the physical world is less than 100 or not.

As illustrated in Table 5, when the system error is $\tau_{s}$, the principles of data selection are described as follows: Using the example in the previous paragraph, in the syntax of “power < “100””, we have to avoid mapping those data that are possibly larger than 100 into query result. So, those data that are more than 95.5 are not mapped to a query result. In the syntax of “power < ?100?”, all possible data that are smaller than 100 have to be mapped into a query result. Hence, all the data that are smaller than 100.5 are mapped into query results. In the syntax of “power < 100”, the value of the sensed data in a query result is simply compared with 100.

### 3.3. Data Aggregation Functions

In an OBEQ, MAX, MIN, SUM, AVG, and GROUP BY, aggregation functions of SQL are also considered. Two syntactic formats in MAX/MIN aggregations are listed below. One restricts the bounded-error of the target column and the other restricts the bounded-error of the aggregated value.

1. SELECT MAX(Data).be(*τ*) FROM SENSORS
2. SELECT MAX(Data.be(*τ*)) FROM SENSORS

The first syntactic example indicates that the maximum value is allowed to have bounded-error *τ*. The second syntactic example indicates that every datum, even the maximum value, is allowed to have bounded-error *τ*. Both syntactic examples return to the same results. Hence, both are executed in the same way. Using the example of “SELECT MAX(Data).be(*τ*) FROM SENSORS”, sensors firstly process raw data with maximum aggregation. After the MAX value is selected, the error of maximum value data is $\tau_{s}$. After query processing has finished, the local results are compressed and sent to the local database. The total error of compressed data becomes ($\tau_{s}+\tau_{c}$). On the local database, the result received from the sensors preserves a data error of ($\tau_{s}+\tau_{c}$), so we can deduce that the compression error for a sensor to use is mentioned above in Equation (8).

The AVG aggregation has two syntactic formats. One restricts the bounded-error of the target column and the other restricts the bounded-error of the aggregated values. Syntactic examples are shown below.

1. SELECT AVG(Data).be(*τ*) FROM SENSORS
2. SELECT AVG(Data.be(*τ*)) FROM SENSORS

The first syntactic format indicates that every average value in a query result is allowed to have bounded-error *τ*. The second syntactic example indicates that every raw datum is allowed to have bounded-error *τ*. In the second syntactic format, after the average logic is operated on a dataset in which each datum has bounded-error *τ*, the bounded-error of the average value would be *τ*. Both syntactic examples return the result with bounded-error *τ*, so both syntactic formats are executed in the same way. The query of “SELECT AVG(power.be(1)) FROM SENSORS” is a demonstration that queries the average power of all the sensor readings. The average value is allowed to have bounded-error 1. Using “SELECT AVG(Data).be(*τ*) FROM SENSORS” as an example, sensors process the average value on raw data and obtain an average value that has system error $\tau_{s}$. After the local query result is generated, it can be compressed with a compression error of $\tau_{c}$. The compressed query result with error ($\tau_{s}$ + $\tau_{c}$) is sent to the local database. Users expect the error of the query result to be τ, so the compression error can be deduced as shown in Equation (8).

The SUM aggregation has two syntactic formats. One restricts the bounded-error of the target column and the other restricts the bounded-error of the aggregated value. Syntactic examples are shown below.

1. SELECT SUM(Data).be(*τ*) FROM SENSORS
2. SELECT SUM(Data.be(*τ*)) FROM SENSORS

The first syntactic format indicates that every summation value in the query result is allowed to have bounded-error *τ*. The second syntactic example indicates that all raw data are allowed to have bounded-error *τ*. In the second syntactic format, after operating the summation logic on a dataset with *n* raw data, the bounded-error of the summation value of the local query result would be *nτ*. Each syntactic format generates different results, so they are executed in a different way.

For example, for “SELECT SUM(power).be(1) FROM SENSORS WHERE city = Taipei” and “SELECT SUM (power.be(1)) FROM SENSORS”, the former one indicates that the bounded-error range for the SUM aggregation value is allowed to be one, and the second indicates that the raw data are allowed to have bounded-error 1. In the example of “SELECT SUM(Data).be(*τ*) FROM SENSORS”, because the query result is merged by multiple local query results, the error of different local query results has to be added up to merge to the final query result. To ensure that the error of the final result is *τ*, each local result can only use part of *τ*. There might be M sensors under an SN. If the quantity of the sensors answering this query can be predicted, this number M can be used. For example, if the user wants to know the summation of the power usage of Taipei City, the old dataset can be used to count the number of sensors in Taipei. If the query cannot be predicted, then each bounded-error sensor can use $\frac{\tau }{M}$ so that the error of the final query result does not fail to meet the user’s expectations. Hence, the local database would rewrite the query instruction before propagating the query to the sensors. If there are *n* raw data in a sensor, the sensor adds up the data and obtains a summation value with system error *n*$\tau_{s}$. It is possible that *n*$\tau_{s}$ is larger than the bounded-error $\frac{\tau }{M}$. If *n*$\tau_{s}$ is larger than $\frac{\tau }{M}$, it means that the system fails to meet the QoS^2^/QoD requirement of the user’s needs. In this case, it is strongly recommended for the users to improve the quality of their hardware. If *n*$\tau_{s}$ does not exceed $\frac{\tau }{M}$, then we use ($\frac{\tau }{M}-n\tau_{s}$) as a compression error $\tau_{c}$ (as shown in Equation (13)) to compress the local query result.

(13) $$\tau_{c}\leq \frac{\tau }{M}-n\tau_{s}$$

In the example of “SELECT SUM(Data.be(*τ*)) FROM SENSORS”, each piece of raw data is allowed to have bounded-error *τ*. If a sensor has N raw data, the summation of these raw data has a bounded-error equal to $n\tau_{s}$. Because each datum is allowed to have bounded-error τ, the summation value is allowed to have bounded-error *n*τ. Hence, the allowed compression error $\tau_{c}$ of this sensor is as per the equation below.

(14) $$\tau_{c}\leq n\tau -n\tau_{s}$$

The query example of “SUM(power).be(1) FROM SENSORS WHERE city = “Taipei”” firstly calculates how much bounded-error from an ED can be allowed. The local database searches the old data and predicts how many nodes execute the query. If we have two nodes in Taipei SN, each node is allowed to have a one out of two bounded-error. Then, the local database changes instructions to “SUM(power).be(0.5)” and sends it to the WSN. Taking “SELECT SUM (power.be(1)) FROM SENSORS” as a demonstration, when an ED receives “SELECT SUM (power.be(1))”, it aggregates 10 pieces of raw data, and the total system errors will be added up to five. Then, the compression error will be five, according to Equation (14). In this case, the bounded-error of the summation value in the local result is 10.

The GROUP BY statement usually comes with aggregation functions. In the aggregation functions, it is common to merge multiple data into one group, which means many local query results from different sensors may have to be merged together. However, in other cases, such as “GROUP BY node”, local query results are not merged. Hence, the group can be divided into two kinds: one needs cross-node aggregation, and the other does not. If a query contains “GROUP BY node”, it is not necessary to send group by tags to an SN or to allocate bounded-error. Multiple local query results can simply be put together and generate the final result without aggregating the groups again. If a query does not contain a “GROUP BY node”, multiple local query results need to be aggregated from different nodes in order to generate the final query result. To merge local query results, group tags must be sent. In the example of query “SELECT SUM(power) FROM SENSORS GROUP BY HOUR(timestamp), DATE(timestamp)”, the database generates the total power consumption each hour every day. Furthermore, the AVG aggregation function needs additional metadata, which are the size of the averaged raw data. In the local database and SN, the result is merged again according to the group tags and metadata. For cross-node merging, bounded-error allocation is calculated as mentioned above in other aggregation functions.

## 4. Evaluation

An OBEQ has benefits in avoiding false alarms, minimizing false-negative rates, and reducing power consumption of WSN wireless nodes to extend the IoT’s system lifetime. To satisfy the demanding QoS^2^/QoD requirements, many potential applications for IoT services can be fostered due to the diversified bounded-error-compliant SQL queries as mentioned above. In this section, we used a wireless watt-meter network to show the benefit of the OBEQ bounded-error query process. In our experiment, our network consisted of 100 EDs, 20 SNs, and 1 sink node. We assumed that the EDs were deployed homogeneously and randomly.

### 4.1. Simulation Scenario

In our experiment, the watt-meter sensor data between January 1st and November 30th had been gathered every day by the offline query using the gather-then-query method. Wireless watt-meter devices were upgraded to support an OBEQ. The compression algorithm [11,14] was trained by the offline data. The rest of the data gathered in December are considered as online data, which were then stored in watt-meters.

Bounded-error query processing starts from writing a query, executing a query, and ends after sending the query result to the local database. How the query instruction is propagated in the WSN is referred to in [11,14]. During the propagation, the query instructions are firstly sent from the local database to the sink, then from the sink to the SNs. After the SN processes the query instructions, the query instructions will be transmitted to the EDs. The technique for each ED in sending data to the local database can be referred to [29]. Each device merges data before transmitting.

**Figure 7:**
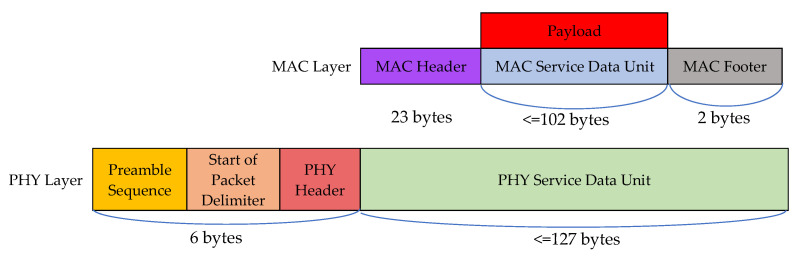
The format of IEEE 802.15.4.

**Figure 8:**
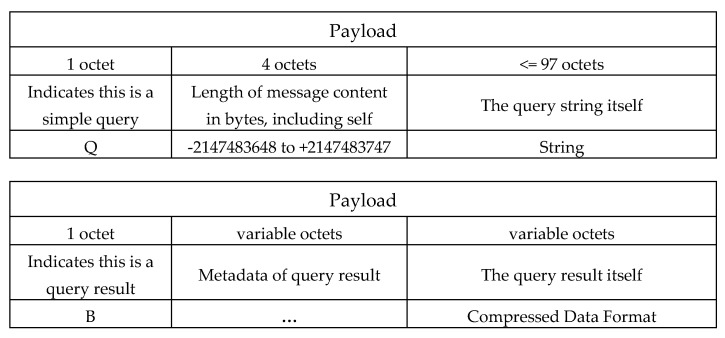
The packet format of the query instruction and query result.

We used the physical layer of IEEE 802.15.4 as our packet format and used the data frame as our MAC layer format. In the MAC layer, the footer format and header format are the same as shown in Figure 7. In previous work, only the compression-related scenarios were considered in the packet format. To cover query scenarios, we embed the format of PostgreSQL [30] in our payload of the MAC layer to cover possible query scenarios. The payload format of the MAC layer is modified from PostgreSQL 8.2. We used the metadata of PostgreSQL, but embed our SQL-like query format and compressed the data format together into the representation of our query instruction and query result. Their packet formats are respectively illustrated in Figure 8.

### 4.2. Evaluation of Power Consumption

In our experiments, the communication cost of the wireless modules is evaluated. Wireless modules are responsible for transmitting data packets. It is assumed that if we use CC2420 as our wireless module, we can then evaluate the communication cost with the parameters from previous work; the parameters are measured by previous work [31] and are shown in Table 6. Using the parameter in this table, we can deduce that it costs 2.08 μJ to receive one bit and 1.83 μJ to transmit one bit.

#### 4.2.1. Query Filter

In this paper, a query filter is proposed to reduce communication costs by analyzing the query instruction. In our experiment, we assumed that an engineer wants to query the power consumption of a specific wireless watt-meter. The query is “SELECT SUM(power.be(1)) FROM SENSORS WHERE _time BETWEEN “2013-12-01 00:00:00” and “2013-12-01 12:00:00””. We consider two scenarios; one simply propagates this query to the entire network and the other uses a query filter.

**Figure 9:**
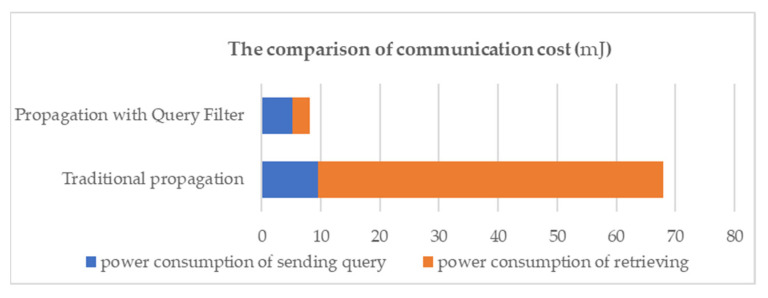
The power consumption of query with and without a query filter.

In this particular case, the query filter reduces 88% of the communication cost, compared to the cost of not using it. Figure 9 illustrates the power consumption of these two scenarios. The darker part represents the power consumption of propagation. The lighter part represents the cost of transmitting the query result. However, if the query result is huge, then the improvement by the query filter will be less significant.

#### 4.2.2. Bounded-Error Query Process

**Figure 10:**
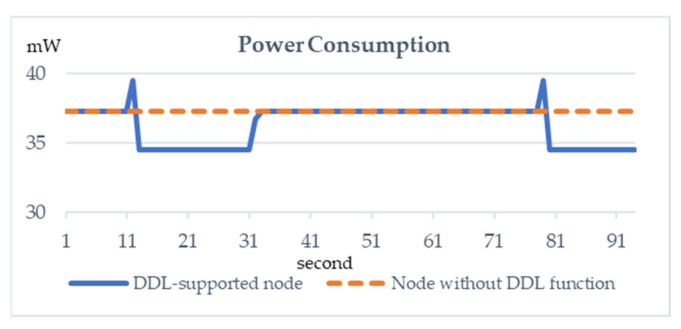
The change in standby power consumption of two nodes.

In this experiment for our OBEQ using DDL, we first compare two nodes; one supports DDL while the other one does not. Data definition instructions are sent to the DDL-supported node at the 13th second and the temperature sensing module was turned off at the 80th second. Another DDL instruction is sent to the DDL-supported node at the 30th second to turn on the temperature sensing module. The instructions for turning off/on the sensor are “ALTER TABLE SENSOR_COLUMN DROP COLUMN temperature” and “ALTER TABLE SENSOR_COLUMN ADD COLUMN temperature”, respectively. We use the LM90 as a temperature sensor, and the power consumption of LM90 is deduced by the datasheet provided by Texas Instruments. In Figure 10, the y-axis represents power consumption and the x-axis represents the time unit in seconds, respectively.

As shown in Figure 10, when the new node receives the packet that carries the DDL instruction, the power consumption increases. When a sensor is turned off, the power consumption of the new node decreases. Our proposed DDL technique can adjust the status of modules in nodes and rearrange the resource of nodes flexibly.

**Figure 11:**
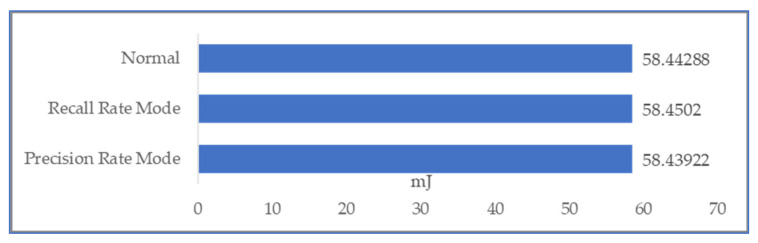
The communication cost of using 3 different strategies.

Then, considering the system error of data, the query result of comparison might be ambiguous. In Section 3.2, we describe three possible approaches to deal with this ambiguity. We assumed that the engineer queries the power consumption whose power usage is more than 1 kWh in December. Each piece of data is allowed to have a 1 Wh system error. We use three different comparison strategies (as shown in Table 5) upon the data and returned 9 results in precision mode, 17 results in recall mode, and 13 results in normal mode. The power consumption comparison is shown in Figure 11. The power consumption of these three modes for DDL is pretty close.

**Figure 12:**
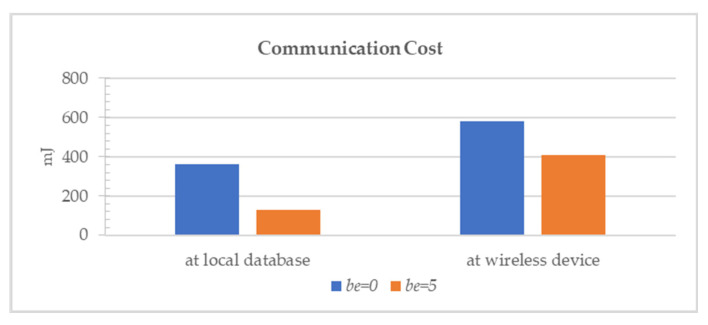
Communication cost of a query with fundamental operations.

In addition, fundamental operations are regularly used functions in SQL queries. Let us say they want to find out the electric fee for each power reading on 1 December 2013 using the query “SELECT (power*5).be(5) FROM SENSORS WHERE time BETWEEN ‘2013-12-01 00:00:00’ AND ‘2013-12-2 00:00:00’”. We assumed the price of electric power was five dollars for 1 kWh. The result is shown in Figure 12 where the y-axis is the communication cost. In our first experiment for fundamental operations, we performed the calculation in the local database. The compression error we used was 0.5 according to Equation (12). An OBEQ reduced 65% of the power consumption compared to the query process that does not support bounded-error queries. In the second experiment for fundamental operations, we performed the calculation in EDs. In this case, the compression error we used was 2.5 according to Equation (10). An OBEQ reduced 30% of performed power consumption compared to the query process that does not support bounded-error queries.

According to the test result from the above OBEQ query instruction, the collected data values of power multiplied by five (aka “power*5”) seldom occur in our training data, so the compression ratio of them with the given compression error is worse than non-calculated data even if we can use larger compression error. However, power data itself can be trained with larger bounded errors within the QoS^2^/QoD requirement to achieve better a compression ratio to further reduce communication costs in our experiment.

**Figure 13:**
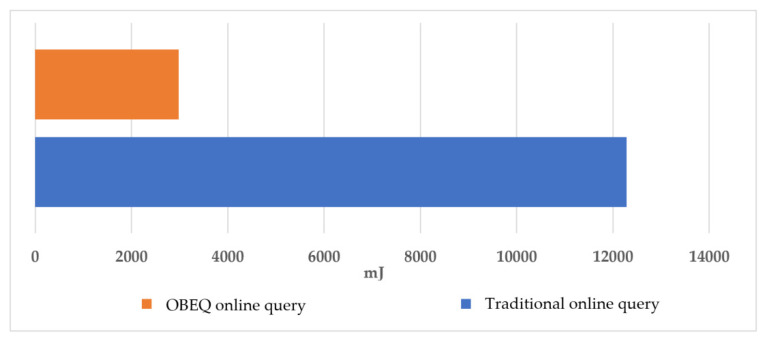
The power consumption of querying all online data.

For the evaluation of gathering all online data using an OBEQ, we assumed that the user wants to observe the power readings on all watt-meters in December. In this evaluation, all the online data were queried by “SELECT power.be(1) FROM SENSORS”. We compared the communication costs of using a traditional online query and an OBEQ with an error restricted to 1 *Wh*. In our evaluation, an OBEQ reduced 76% of the communication cost caused by transmitting the query result. In Figure 13, the lighter one showed the power consumption of the OBEQ online query process using bounded-error compression and the other showed the power consumption of the traditional online query process.

Moreover, we evaluated the communication cost by retrieving arbitrary data from arbitrary EDs. In the first experiment, we randomly retrieved one thousand pieces of data from arbitrary wireless devices and recorded their power consumption one thousand times. In the second experiment, we randomly retrieved two thousand pieces of data from arbitrary wireless devices and recorded their power consumption one thousand times, etc. In the final experiment, we randomly retrieve ten thousand pieces of data from arbitrary wireless devices and recorded their power consumption one thousand times. In each case, we compared the difference in power consumption using bounded-error 1 and lossless compression.

**Figure 14:**
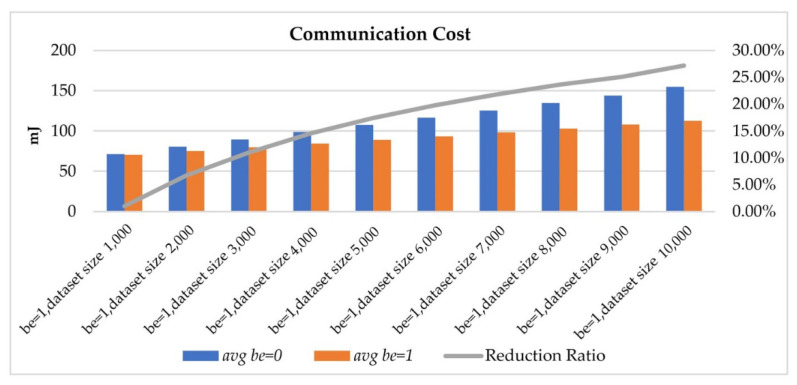
The power consumption of querying different-size datasets.

In Figure 14, we illustrate the power consumption of transmitting datasets in different sizes. Under the query condition that the bounded-error was one, transmitting different sizes of the dataset spent 70.31 to 112.61 mJ on average for communication costs with a bounded-error of 1 Wh, while the traditional query process spent 71.03 to 154.66 mJ. We further evaluated the reduction ratio of power consumption in transmitting different sizes of the dataset. This evaluation shows that an OBEQ can successfully reduce the power consumption by up to 27%, as shown in the gray curve of Figure 14.

#### 4.2.3. Bounded-Error Data Aggregation

**Figure 15:**
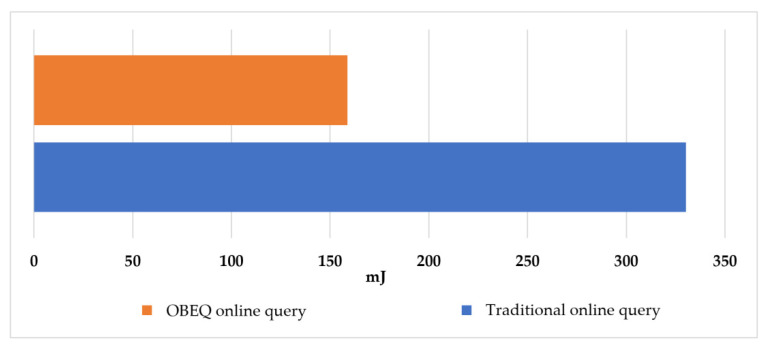
The power consumption of MAX aggregation.

To evaluate the MAX aggregation function for an OBEQ, we assumed that the user wants to find out the maximum power usage per hour of each house between December 1st 2013 and December 7th 2013, using “SELECT MAX(power).be(1) FROM SENSORS WHERE time BETWEEN ‘2013-12-01 00:00:00’ AND ‘2013-12-7 0:00:00’ GROUP BY DATE(time), HOUR(time), node”. In this evaluation for the MAX aggregation function applied in an OBEQ, as shown in Figure 15, we save more than 51% of the power consumption during the bounded-error query process when compared to the traditional query process that only supports lossless compression.

**Figure 16:**
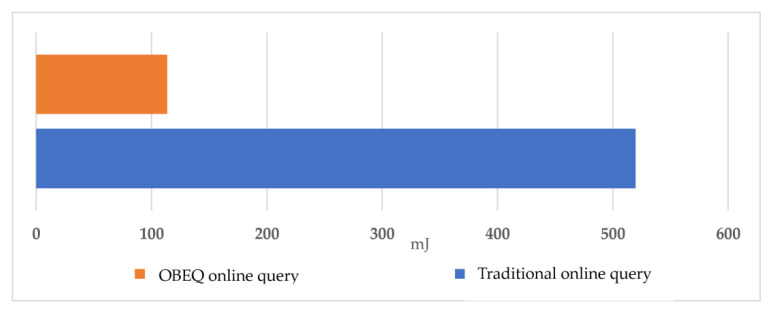
The power consumption of AVG aggregation.

Again, we assumed that our user wants to find out the average power usage per hour of each house between December 1st 2013 and December 7th 2013 using “SELECT AVG(power).be(1) FROM SENSORS WHERE _time BETWEEN ‘2013-12-01 00:00:00’ AND ‘2013-12-14 0:00:00’ GROUP BY DATE(_time), HOUR(_time), node”. The query results from the AVG aggregation function were firstly aggregated and compressed in wireless watt-meters and then sent to SNs. SNs aggregated and compressed the data again and then sent the query result to the local database. In Figure 16 of this evaluation for the AVG aggregation function, we save about 78% of the power consumption by an OBEQ.

**Figure 17:**
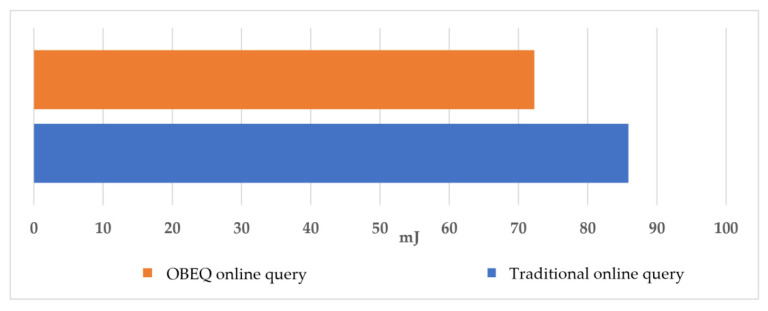
The power consumption of SUM aggregation.

Finally, we assumed that the user wants to find out the summation of power usage per hour on 1 December 2013 using “SELECT SUM(power).be(10) FROM SENSORS WHERE _time = 2013-12-1’ GROUP BY HOUR(_time), node”. The intention of this SUM aggregation query was to observe how the power usage changes over time, so we did not need to be extremely accurate. In this case, we used bounded-error τ equal to 10 to compress the query result. The query results were firstly aggregated into SUM without violating the total bounded error of 10, compressed in wireless watt-meters, and then sent to SN. SN aggregated and compressed the data again and sent the query result to the local database. As shown in Figure 17 for the SUM aggregation evaluation, we save more than 15.78% of the power consumption compared to the traditional online query without the bounded-error query process.

**Figure 18:**
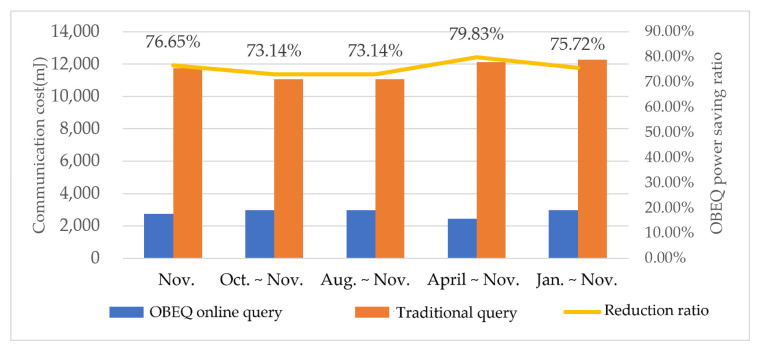
The power consumption of different-size training data.

Since the proposed OBEQ scheme applies data compression algorithms from previous work [14,15]. For better data compression performance to reduce communication costs, the corresponding settings and parameters (e.g., codebooks) in data compression were trained by historical offline data. We simply conducted the experiments for communication costs of different sizes in a historical training dataset. We used the “SELECT power.be(1) FROM SENSORS” query to find out the communication cost from different sizes of historical training data. As shown in Figure 18, it indicates that if the historical training data size is not that large, an OBEQ can still effectively reduce the communication cost for energy-efficient IoT sensors.

From an OBEQ performance evaluation perspective, the calculations including data compressions about the change in bounded errors at each step online during the query process were all performed by the CPU in sensors. As the power consumption of transmitting one-bit data on a WSN is higher than that of computing a thousand lines of code on a CPU of sensors, our OBEQ evaluations for online queries indicate that applied bounded-error compression can save more power consumption. Moreover, in the three-tier WSN architecture (as shown in Figure 2) of OBEQs, all the nodes on the same tier can calculate and transmit data in parallel. Thus, OBEQs will not suffer more latency time costs than traditional online queries.

## 5. Conclusions and Future Works

In this paper, edge-computing of an OBEQ scheme for energy-efficient IoT sensors is proposed. Unlike other previous query process methods, system errors during the query process are considered, and our query process technique even allows users to restrict data errors at certain levels during queries. Most important of all, the bounded-error techniques are used to reduce communication costs. In our experiments, our OBEQ can efficiently reduce communication costs. The reduction rate is related to the query result and the size of the query result. In our experiment, communication costs are reduced by up to 88%.

In the near future, many techniques can be proposed based on our query scheme. For example, adaptive codebook techniques can be applied to our OBEQ system to achieve better a compression ratio more flexibly, and data mining techniques can be developed based on bounded-error data, or error bound can be deduced with mathematical analysis techniques to reduce system errors before compression, etc.

## Figures and Tables

**Table 1:** An instance sample of ED_CLUSTER_METADATA.

Node_ID	Coordinate	Time	Type	Columns
RS02000D6F000172B951	(12,14)	3 January 2020	watt-meter	power, current, voltage
RS02000D6F000172BC59	(13,12)	12 July 2020	water-meter	water usage, water pressure
RS02000D6F000172C822	(15,13)	21 September 2020	watt-meter	power, current, voltage

**Table 2:** SENSOR_COLUMN table.

Node_ID	Current	Voltage	Timestamp
RS02000D6F000072EE0E	True	True	False
RS02000D6F000072F666	True	False	True
RS02000D6F000072F737	False	True	True

**Table 3:** An example of the MODULE_COLUMN table.

Module	Column	System_Error
Ammeter	current	0.1 A
Voltmeter	voltage	1.5 V
Watt-meter	power	0.5 Wh

**Table 4:** An example of data.

Data Type	Value	Meaning of Value
Raw data	116	Voltage value collected by sensors with system error applied
Physical data	116.5	Voltage value in reality
Query result	117	Decoded data presented to the user with system error and specified bounded-error

**Table 5:** The comparison syntax table in different query strategies of an OBEQ.

Logic	Syntax	Actual Process
Larger than	data>“value”	Raw data must be larger than value + $\tau_{s}$
	data>?value?	Raw data must be larger than value − $\tau_{s}$
	data>value	Raw data must be larger than the value
Smaller than	data<“value”	Raw data must be smaller than value − $\tau_{s}$
	data<?value?	Raw data must be smaller than value + $\tau_{s}$
	data<value	Raw data must be smaller than the value

**Table 6:** The power consumption of wireless module.

Parameter	Value
$I_{Tx}$	17.4 mA
$I_{Rx}$	19.7 mA
$T_{Tx}$	3.2 × 10^−5^ s
$T_{Rx}$	3.2 × 10^−5^ s
$V_{Tx}$	3.3 V
$V_{Rx}$	3.3 V
